# The Triglyceride‐Glucose (TyG) Index and Incident Diabetes and Cardiovascular Events in People Living With HIV‐1 Infection. A 14‐Year Observational Cohort Study

**DOI:** 10.1111/eci.70258

**Published:** 2026-09-02

**Authors:** Daniele Pastori, Gianluca Gazzaniga, Tommaso Brogi, Marco Rivano Capparuccia, Caterina Fimiani, Claudio Mastroianni, Ivano Mezzaroma

**Affiliations:** ^1^ Department of Medical and Cardiovascular Sciences Sapienza University of Rome Rome Italy; ^2^ IRCCS Neuromed, Località Camerelle Pozzilli Isernia Italy; ^3^ Department of General Surgery, Surgical Specialty and Anesthesiology Paride Stefanini Sapienza University of Rome Rome Italy; ^4^ Department of Internal Medicine and Medical Specialties Sapienza University of Rome Rome Italy; ^5^ Department Internal Medicine Endocrine‐Metabolic Sciences and Infectious Diseases, Policlinico Umberto I Rome Italy; ^6^ Department of Public Health and Infectious Diseases Sapienza University of Rome Rome Italy; ^7^ Department of Translational and Precision Medicine Sapienza University of Rome Rome Italy

**Keywords:** cardiovascular disease, diabetes, HIV, TyG index

## Abstract

**Background:**

People living with HIV (PWH) are at increased risk for metabolic and cardiovascular diseases. However, there is no structured prevention strategy for these subjects. The triglyceride‐glucose (TyG) index has shown predictive value for Type 2 diabetes mellitus (T2DM) and cardiovascular events (CVEs) in the general population, but its utility in PWH remains unclear.

**Methods:**

We conducted a retrospective study including 477 PWH without baseline T2DM. TyG index was calculated as follows: ln[fasting triglycerides (mg/dL) × fasting glucose (mg/dL)]/2. ROC analysis was used to identify the optimal TyG threshold for T2DM, and logistic regression analysis was used to evaluate the association with T2DM. The association between TyG index and CVEs was investigated using Kaplan–Meier survival analysis and Cox proportional hazards regression models.

**Results:**

477 PWH were included. Median age was 38 years and 26% were women. During a median follow‐up of 14.2 years, 38 participants developed T2DM and 45 experienced CVEs. The TyG index demonstrated fair discrimination for incident T2DM (AUC: 0.675, 95% CI 0.591–0.759), with an optimal threshold of 4.78. Participants with TyG ≥ 4.78 had a higher risk of developing T2DM (aOR 2.86, 95% CI 1.35–6.12). PWH in the highest TyG tertile had also higher risk for CVEs compared with those in the lowest tertile (aHR 3.31, 95% CI 1.10–9.97).

**Conclusion:**

In PWH, a higher TyG index was independently associated with an increased risk of both T2DM and CVEs over long‐term follow‐up. The TyG index represents a practical, low‐cost tool for early metabolic risk stratification in this population.

## Graphical abstract

People living with HIV (PWH) face an increased risk of Type 2 diabetes mellitus (T2DM) and cardiovascular events (CVEs), highlighting the need for simple risk stratification tools. In this retrospective study of 477 PWH followed for 14.2 years, a higher triglyceride‐glucose (TyG) index independently predicted incident T2DM and CVEs. A TyG threshold of 4.78 identified individuals at increased diabetes risk. The TyG index is an inexpensive, readily available biomarker that may support early metabolic and cardiovascular risk assessment in PWH.

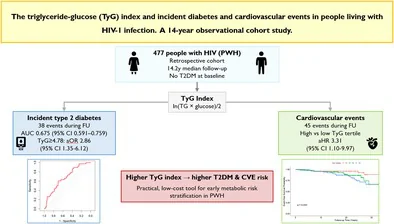

## 1 Introduction

Antiretroviral therapy (ART) has remarkably reduced acquired immunodeficiency syndrome (AIDS)‐related mortality in people living with human immunodeficiency virus (HIV) (PWH), extending life expectancy to levels comparable with the general population.

However, non‐AIDS‐related causes including metabolic and chronic diseases have emerged as new challenges, with cardiovascular disease (CVD) representing a major contributor to mortality risk [1]. Indeed, the relative risk of CVD among this population was 2.16 compared to non‐HIV infections [1]. This elevated risk of CVD is multifactorial. First, traditional CV risk factors are prevalent in this population, with dyslipidemia and smoking each affecting over 30% of individuals, and hypertension being present in approximately 20% [2, 3]. Notably, this excess CV risk is driven also by chronic systemic inflammation and immune activation related to HIV infection itself [4] and the metabolic side effects of long‐term ART [5, 6, 7]; these factors contribute substantially to the development of atherosclerosis and cardiovascular events (CVEs) in this group. Notably, currently recommended risk stratification tools underestimate CV risk in PWH [8].

Additionally, PWH are at higher risk of developing insulin resistance and Type 2 diabetes mellitus (T2DM) compared to the general population, with cumulative incidence up to 5.9 per 1000 person‐years [9]. T2DM in this population further amplifies cardiovascular morbidity and mortality. Consequently, early identification of individuals at higher risk for T2DM is critical to enable timely preventive strategies and targeted interventions, and screening at baseline and after ART initiation through glycated haemoglobin (A1C) and blood glucose check is recommended [10].

To this aim, the use of an easy non‐invasive index would be useful for T2DM risk stratification. Among the others, the triglyceride‐glucose (TyG) index has emerged as a simple and reliable surrogate marker of insulin resistance. Unlike more complex methods such as the hyperinsulinemic‐euglycemic clamp or the homeostasis model assessment of insulin resistance (HOMA‐IR), the TyG index is calculated using only fasting triglyceride and glucose levels, which are routinely measured in clinical practice, and has provided comparable results to the other more complex methods [11]. In the general population, a higher TyG index has been strongly associated with incident T2DM and CVEs [12], supporting its potential as a practical tool for metabolic risk stratification [13, 14].

Despite the growing evidence supporting the TyG index as a marker of insulin resistance and a predictor of T2DM in the general population, data regarding its role in PWH remain limited. To date, no specific thresholds for the TyG index have been validated in this population, which may differ metabolically due to chronic inflammation and long‐term ART exposure.

The primary aim of this study was to investigate the association between baseline TyG index and incident T2DM in a cohort of PWH; in particular, we sought to identify a potential TyG index threshold that could serve as a practical tool to guide individualized risk stratification and inform early preventive strategies in clinical practice. Secondly, we investigated the association between TyG index and the risk for CVEs during follow‐up.

## 2 Methods

### 2.1 Study Design and Inclusion Criteria

PWH were recruited from the Infectious Diseases Outpatient Clinic at Policlinico Umberto I, Rome, Italy, until 2022. At the initial assessment, demographic data, detailed medical history, and laboratory parameters were systematically collected. Subjects were prescribed ART according to European Guidelines [15].

### 2.2 Inclusion and Exclusion Criteria

Eligible participants met the following inclusion criteria: confirmed HIV‐1 infection with ongoing effective ART, age ≥ 18 years, and provision of written informed consent.

Subjects were excluded if they had a diagnosis of T2DM at baseline. T2DM was defined according to the following criteria: a random plasma glucose ≥ 200 mg/dL (11.1 mmol/L), fasting plasma glucose ≥ 126 mg/dL (7.0 mmol/L), plasma glucose ≥ 200 mg/dL (11.1 mmol/L) measured 2 h after an oral glucose tolerance test, or A1C ≥ 6.5% (48 mmol/mol) [16].

The study was approved by the competent Institutional Review Board. All participants provided written informed consent before inclusion. The study procedures conformed to the ethical standards of the 1975 Declaration of Helsinki and its later revisions.

### 2.3 Baseline Features

Baseline variables included demographic characteristics and clinical data relevant to HIV infection and cardiovascular risk assessment. Specifically, data on sex, age, body mass index (BMI, kg/m^2^) were collected. Virological and immunological markers included hepatitis C virus (HCV) and hepatitis B virus (HBV) coinfection status, CD4+ T lymphocyte nadir (cells/μL), CD4+ T lymphocyte percentage, absolute CD4+ T lymphocyte count (cells/μL), and duration of ART.

Established cardiovascular risk factors, including hypertension, dyslipidemia, chronic obstructive pulmonary disease (COPD), and history of stroke or transient ischemic attack (TIA) were recorded. Hypertension and dyslipidemia were defined by elevated blood pressure or lipid levels, respectively, and/or current use of antihypertensive or lipid‐lowering medication. A history of coronary heart disease (CHD), defined as prior myocardial infarction (MI) or documented coronary artery disease, was also noted.

Routine laboratory parameters included total cholesterol, serum creatinine, haemoglobin, platelet count, and white blood cell count. Hepatic function markers, including aspartate aminotransferase (AST), alanine aminotransferase (ALT), and gamma‐glutamyl transferase (GGT), were also assessed.

### 2.4 TyG Index and Cardiovascular Event Definitions

For each patient, TyG index was calculated using baseline values for triglycerides and fasting glucose. Specifically, this formula was used [17, 18]:

$$\mathrm{TyG}\;\text{index}=\ln\left[\text{fasting triglycerides}\;\left(\mathrm{mg}/\mathrm{dL}\right)\times \text{fasting glucose}\;\left(\mathrm{mg}/\mathrm{dL}\right)\right]/2.$$

CVEs were defined as fatal or non‐fatal ischemic stroke, transient ischemic attack, fatal or non‐fatal myocardial infarction, or coronary revascularization. All events were actively monitored throughout follow‐up.

### 2.5 Statistical Analysis

Descriptive analyses were conducted to summarize the baseline characteristics of the study cohort. The distribution of continuous variables was assessed using the Kolmogorov–Smirnov test. Continuous variables exhibiting a normal distribution were presented as means with standard deviations (SD), whereas those with a non‐normal distribution were reported as medians and interquartile ranges (IQR). Categorical variables were described using absolute numbers and corresponding percentages. For comparisons between groups, the independent samples *t*‐test was applied for normally distributed continuous variables, while the Wilcoxon rank‐sum test was used for non‐normally distributed continuous variables. Differences in categorical variables were evaluated using the chi‐square test or Fisher's exact test, as appropriate.

To assess the predictive performance of the TyG index for incident T2DM during follow‐up, a receiver operating characteristic (ROC) curve analysis was conducted, treating the TyG index as a continuous variable. The area under the ROC curve (AUC) along with its 95% confidence interval (CI) was calculated to evaluate the overall discriminatory ability of the TyG index in identifying individuals who developed T2DM. The optimal cut‐off value for the TyG index was determined using the Youden index, defined as the point maximizing the sum of sensitivity and specificity (Youden index = sensitivity + specificity − 1). Sensitivity and specificity corresponding to this optimal threshold were also reported.

To examine the association between baseline variables and the incidence of T2DM during follow‐up, logistic regression analyses were performed. The TyG index was categorized according to the optimal cut‐off value derived from the ROC curve analysis (i.e., below vs. above the identified threshold). Initially, univariable logistic regression analyses were conducted to assess the individual association of each baseline variable with incident T2DM. Subsequently, clinically relevant variables that showed a significant association in the univariable analyses were included in a multivariable model to evaluate their independent association with T2DM incidence. Results were expressed as odds ratios (OR) with corresponding 95% CI.

Consequently, we investigated the association between TyG index and CVEs. The cumulative incidence of CVEs was first estimated using Kaplan–Meier survival curves, and differences between groups (according to tertiles) were assessed using the log‐rank test. Pairwise comparisons were performed using Holm‐adjusted log‐rank tests. A test for linear trend across tertiles was also performed. Subsequently, Cox proportional hazards regression models were employed to further evaluate the association between the TyG index and the risk of incident CVEs.

In the univariable model, the TyG index (categorized in tertiles) was examined as the primary predictor. A multivariable Cox regression model including clinically relevant demographic covariates, namely age and BMI, was further fitted. The results were presented as hazard ratios (HR) with corresponding 95% CI. The proportional hazards assumption was evaluated using Schoenfeld residuals and found to be satisfied.

Data analysis was conducted using R Software (version 4.2.3; R Foundation for Statistical Computing, Vienna, Austria), with a two‐tailed significance level of *α* = 0.05 for all tests.

## 3 Results

A total of 477 PWH were included in the analysis, of whom 126 (26%) were women, with a median age of 38.0 years (IQR 31.8–47.8). Overall, 38 cases of incident T2DM were observed over a median follow‐up of 14.2 (6.3–22.6) years.

### 3.1 Descriptive Analysis

Baseline characteristics of the population are summarized in Table 1. Among the 477 participants included in the analysis, 155 (32%) had a TyG index above the ROC‐derived threshold (see next paragraph for details). Participants with a higher TyG index had a longer duration of ART (median 23.0 vs. 18.0 years, *p* < 0.001). A higher prevalence of dyslipidemia (40% vs. 7.5%, *p* < 0.001) and elevated levels of total cholesterol and GGT were also observed in the high TyG group (all *p* < 0.001). Furthermore, individuals with a higher TyG index had lower CD4+ T lymphocytes at nadir counts (median 187.5 vs. 224.0 cells/μL, *p* = 0.028) and were more frequently coinfected with HCV (30% vs. 20%, *p* = 0.016).

**TABLE 1:** Subjects' demographic and clinical characteristics stratified by TyG index category (below vs. above the ROC‐derived threshold).

Characteristic	*N*	Overall, *N* = 477	TyG index < 4.78, *N* = 322	TyG index ≥ 4.78, *N* = 155	*p*
Female Sex	477	126 (26%)	94 (29%)	32 (21%)	0.047
Age at enrolment (years)	477	38.0 (31.8, 47.8)	37.3 (31.3, 46.7)	38.9 (32.7, 49.6)	0.121
Years on ART	457	19.0 (11.0, 25.0)	18.0 (9.0, 24.0)	23.0 (15.0, 27.0)	< 0.001
HCV coinfection	469	110 (23%)	64 (20%)	46 (30%)	0.016
HBV coinfection	470	64 (14%)	48 (15%)	16 (10%)	0.165
CD4 nadir (cells/μL)	441	217.0 (111.0, 345.0)	224.0 (125.0, 350.0)	187.5 (95.5, 321.3)	0.028
BMI (kg/m^2^)	424	23.7 (22.0, 25.8)	23.5 (21.9, 25.4)	24.1 (22.1, 26.3)	0.130
Hypertension	477	20 (4.2%)	17 (5.3%)	3 (1.9%)	0.088
Dyslipidemia	477	86 (18%)	24 (7.5%)	62 (40%)	< 0.001
COPD	477	1 (0.2%)	1 (0.3%)	0 (0%)	> 0.999
History of stroke or TIA	476	3 (0.6%)	2 (0.6%)	1 (0.6%)	> 0.999
History of MI or CHD	477	7 (1.5%)	5 (1.6%)	2 (1.3%)	> 0.999
Fasting glucose (mg/dL)	477	88.1 (82.2, 95.5)	86.1 (81.1, 93.2)	92.1 (85.0, 99.1)	< 0.001
Total cholesterol (mmol/L)	459	4.5 (3.8, 5.4)	4.3 (3.6, 5.2)	5.0 (4.2, 6.0)	< 0.001
Triglycerides (mg/dL)	477	119.5 (85.0, 172.6)	98.2 (74.3, 121.2)	208.8 (173.0, 269.9)	< 0.001
Creatinine (mg/dL)	473	0.9 (0.8, 1.0)	0.9 (0.8, 1.0)	0.9 (0.8, 1.1)	0.577
Haemoglobin (g/dL)	395	14.3 (13.3, 15.1)	14.1 (13.1, 15.0)	14.6 (13.6, 15.4)	0.008
Platelet count (10^9^/L)	395	206.0 (169.0, 244.0)	204.0 (168.0, 241.0)	207.5 (170.3, 249.3)	0.574
White blood cell count (10^9^/L)	396	5.59 (4.60, 6.88)	5.48 (4.43, 6.81)	5.97 (4.92, 6.93)	0.066
CD4+ T lymphocytes (%)	395	23.0 (14.0, 31.0)	23.0 (15.0, 31.0)	22.5 (13.0, 30.0)	0.297
CD4+ T lymphocytes (cells/μL)	395	413.0 (238.0, 623.0)	422.0 (246.0, 622.0)	402.0 (230.8, 648.8)	0.796
AST (U/L)	393	25.0 (19.0, 35.0)	25.0 (19.0, 34.0)	25.0 (20.0, 39.0)	0.860
ALT (U/L)	393	24.0 (16.0, 38.0)	24.0 (16.0, 39.3)	24.0 (16.0, 37.0)	0.738
GGT (U/L)	371	24.0 (16.0, 45.5)	22.0 (14.0, 39.0)	30.5 (20.3, 56.0)	< 0.001

Abbreviations: ALT, alanine aminotransferase; ART, antiretroviral therapy; AST, aspartate aminotransferase; BMI, body mass index; CHD, coronary heart disease; COPD, chronic obstructive pulmonary disease; GGT, gamma‐glutamyl transferase; HBV, hepatitis B virus; HCV, hepatitis C virus; MI, Myocardial Infarction; TIA, transient ischemic attack; TyG, triglyceride‐glucose index.

### 3.2 Incident T2DM

**FIGURE 1:**
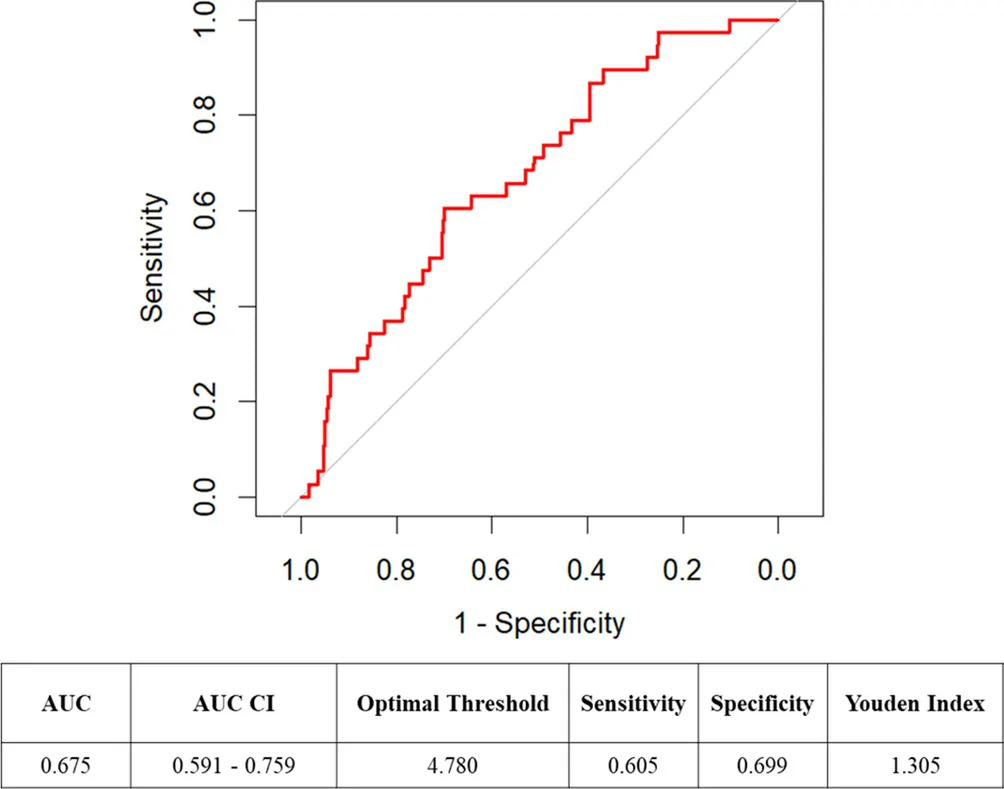
ROC curve of the TyG index for predicting incident T2DM during follow‐up. AUC, Area Under the Curve; CI, Confidence Interval.

Baseline characteristics stratified for incident T2DM (*n* = 38) are reported in Table S1. In the ROC curve analysis assessing the ability of the TyG index to predict incident T2DM during follow‐up (Figure 1), the TyG index showed a fair discriminative performance, with an AUC of 0.675 (95% CI: 0.591–0.759, *p* < 0.001). The optimal cut‐off value determined using the Youden index was 4.78. At this threshold, the TyG index achieved a sensitivity of 61% and a specificity of 70% for identifying individuals who developed T2DM.

Table 2 shows the results of univariable and multivariable logistic regression analyses assessing the association between baseline variables and the risk of incident T2DM during follow‐up. In the univariable analysis, higher TyG index values as a continuous variable were strongly associated with increased odds of developing T2DM (OR 5.96, 95% CI: 2.12–16.9, *p* < 0.001). When analysed as a categorical variable (high vs. low TyG group, based on the ROC‐derived cut‐off), the high TyG group also showed a significant association with incident T2DM (OR 3.57, 95% CI: 1.82–7.19, *p* < 0.001). Other factors significantly associated with T2DM in univariable models included older age (OR 1.21, 95% CI: 1.06–1.39, *p* = 0.005 per 5‐years increase), longer duration on ART (OR 1.33, 95% CI: 1.09–1.66 per 5‐years increase, *p* = 0.007), and higher BMI (OR 1.14, 95% CI: 1.05–1.24, *p* = 0.002).

**TABLE 2:** Univariable and multivariable logistic regression models for predictors of incident T2DM.

Characteristic	Univariable model			Multivariable model		
	OR	95% CI	*p*	OR	95% CI	*p*
High TyG group (vs low)^a^	3.57	1.82, 7.19	< 0.001	2.86	1.35, 6.12	0.006
Male Sex (vs Female)	1.65	0.75, 4.16	0.248	—	—	—
Age (per 5‐years increase)	1.21	1.06, 1.39	0.005	1.13	0.96, 1.32	0.143
Years on ART (per 5‐years increase)	1.33	1.09, 1.66	0.007	—	—	—
HCV coinfection	0.86	0.36, 1.85	0.716	—	—	—
HBV coinfection	0.73	0.21, 1.91	0.564	—	—	—
CD4 nadir (above vs. below median)	0.57	0.27, 1.14	0.120	—	—	—
BMI	1.14	1.05, 1.24	0.002	1.11	1.02, 1.22	0.027
Hypertension	1.3	0.20, 4.75	0.732	—	—	—
Dyslipidemia	1.23	0.51, 2.68	0.614	—	—	—
Total cholesterol (mmol/L)	0.94	0.71, 1.21	0.627	—	—	—
Platelet count (10^9^/L)	1.00	0.99, 1.00	0.150	—	—	—
White blood cell count (10^9^/L)	1.00	1.00, 1.00	0.334	—	—	—
CD4+ T lymphocytes (cells/μL)	1.00	1.00, 1.00	0.303	—	—	—

Abbreviations: ART, antiretroviral therapy; BMI, body mass index; CI, Confidence Interval; HBV, hepatitis B virus; HCV, hepatitis C virus; OR, Odds Ratio; TyG, triglyceride‐glucose index.

^a^ Subject were stratified according to optimal threshold from ROC analysis (TyG ≥ 4.78); age and years on ART were modelled as continuous variables and scaled to represent OR per 5‐year increase.

In the multivariable model, which included the high TyG group, age, and BMI, belonging to the high TyG group remained independently associated with an increased risk of incident T2DM (OR 2.86, 95% CI: 1.35–6.12, *p* = 0.006). Higher BMI (OR 1.11, 95% CI: 1.02–1.22, *p* = 0.027) was also confirmed as a significant independent predictor, while age was not. To further account for potential confounding, an additional multivariable model was constructed including years on ART instead of age (Table S2). In this alternative model, being in the high TyG group remained a significant independent factor associated with an increased risk of T2DM, further supporting the robustness of this association.

### 3.3 Cardiovascular Events

**FIGURE 2:**
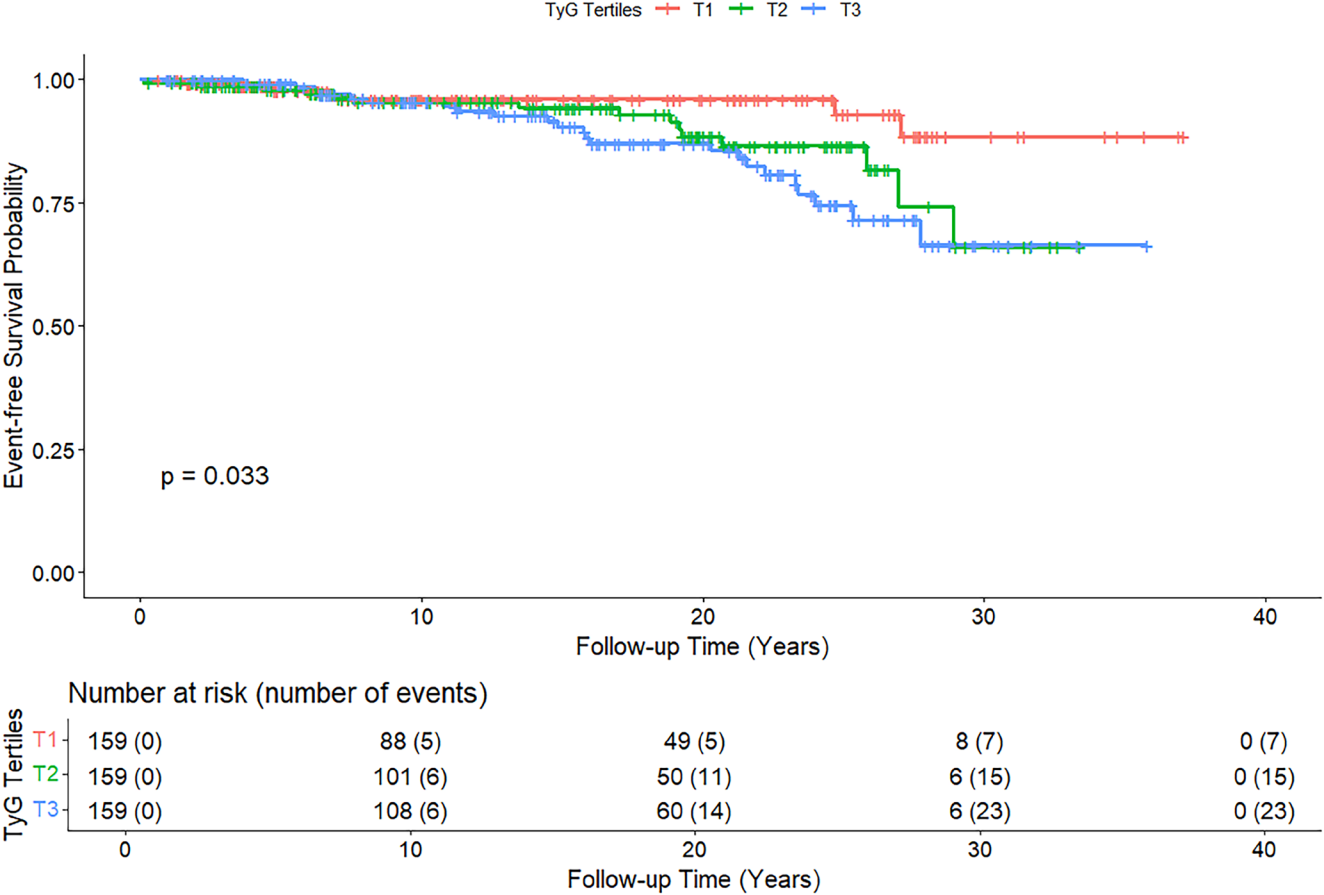
Kaplan–Meier curves for vascular events stratified by TyG index tertiles. Reported *p*‐value corresponds to the overall log‐rank test.

Overall, 45 CVEs occurred during the follow‐up period, with an overall incidence rate of 6.4 per 1000 person‐years. Baseline characteristics of the study population, stratified by incident CVEs, are reported in Table S3. When modelled as a continuous variable in univariable Cox regression analysis, the TyG index was significantly associated with CVE risk (HR 3.27, 95% CI 1.35–7.92; *p* = 0.009). The study population was subsequently divided into tertiles according to TyG values. Kaplan–Meier analysis (Figure 2) demonstrated a significant difference in CVE‐free survival across TyG tertiles (overall log‐rank *p* = 0.033). Pairwise comparisons showed a significant difference between T1 and T3 after Holm adjustment (adjusted *p* = 0.028), while increasing TyG tertile was associated with higher CVE risk (*p* for trend = 0.010).

In univariable Cox regression analyses, PWH in TyG T3 had a significantly higher risk of CVE (HR 2.96, 95% CI 1.27–6.90; *p* = 0.012) compared with TyG T1 (Table 3). In the multivariable Cox regression model, TyG T3 remained independently associated with an increased risk of CVE compared with TyG T1 (HR 3.31, 95% CI 1.10–9.97; *p* = 0.033) after adjustment for age and BMI.

**TABLE 3:** Univariable and multivariable Cox regression model for CVE risk.

Characteristic	Univariable model			Multivariable model		
	HR	95% CI	*p*	HR	95% CI	*p*
TyG T2 (vs T1)	2.17	0.88, 5.34	0.091	2.79	0.88, 8.83	0.080
TyG T3 (vs T1)	2.96	1.27, 6.90	0.012	3.31	1.10, 9.97	0.033
Male Sex (vs Female)	2.12	0.99, 4.57	0.054	—	—	—
Age (per 5‐years increase)	1.39	1.22, 1.59	< 0.001	1.36	1.13, 1.64	0.001
Years on ART (per 5‐years increase)	0.83	0.65, 1.07	0.160	—	—	—
HCV coinfection	0.72	0.36, 1.47	0.372	—	—	—
HBV coinfection	0.60	0.22, 1.69	0.335	—	—	—
CD4 nadir (above vs. below median)	0.72	0.37, 1.39	0.326	—	—	—
BMI	1.05	0.95, 1.16	0.356	1.01	0.92, 1.12	0.770
Hypertension	8.55	3.28, 22.3	< 0.001	—	—	—
Dyslipidemia	2.22	1.18, 4.18	0.013	—	—	—
Total cholesterol (mmol/L)	1.00	0.79, 1.26	0.992	—	—	—
Platelet count (10^9^/L)	1.00	1.00, 1.00	0.731	—	—	—
White blood cell count (10^9^/L)	1.00	1.00, 1.00	0.589	—	—	—
CD4+ T lymphocytes (cells/μL)	1.00	1.00, 1.00	0.209	—	—	—

*Note:* Age and years on ART were modelled as continuous variables and scaled to represent HR per 5‐year increase.

Abbreviations: ART, antiretroviral therapy; BMI, body mass index; CI, Confidence Interval; HBV, hepatitis B virus; HCV, hepatitis C virus; HR, Hazard Ratio; TyG, triglyceride‐glucose index.

## 4 Discussion

In this longitudinal study of young PWH, we found that a higher TyG index at baseline was significantly associated with an increased risk of incident T2DM and CVEs during a long‐term follow‐up.

The TyG index demonstrated a good discriminatory ability for predicting future T2DM, with an optimal cut‐off value of ≥ 4.78 identified through ROC curve analysis. Individuals in the high TyG group had a nearly 3‐fold higher risk of developing T2DM, even after adjustment for key confounders such as age and BMI. These variables were included given their well‐established association with T2DM risk [19, 20], and the model results indicate that the association between elevated TyG values and T2DM risk remained independent of both age and adiposity. Importantly, this association remained robust also after further adjustment for duration of ART, which has well‐known metabolic effects [21].

Our findings confirm also in PWH a growing body of evidence supporting the TyG index as a surrogate marker of insulin resistance and a predictor of adverse metabolic and cardiovascular outcomes in the general population [12]. Very few studies investigated the role of TyG in PWH with shorter follow‐up and none of them from European countries. Wei et al., in a cohort of PWH from Southwest China, reported that high TyG index trajectories were associated with a 2.37‐fold increased risk of incident T2DM, a finding that is consistent with our results. Notably, participants in our cohort had substantially longer exposure to ART, with a median ART duration of 19 years, whereas only 9.2% of individuals in the Wei et al. cohort had more than 10 years of ART exposure. This difference may have contributed to further alterations in the metabolic profile of people with HIV in our study [22]. However, the TyG threshold associated with increased T2DM risk in the study by Wei et al. appeared substantially different from that identified in our cohort (> 8.7 vs. 4.78). A comparable threshold (8.8) was reported by Unger and colleagues in a cross‐sectional study of 525 adults from Argentina for the discrimination of metabolic syndrome [23]. Importantly, these thresholds are not directly comparable with the one we identified in our study, as both Wei et al. and Unger et al. used a different formula for calculating the TyG index, namely [ln(TG [mg/dL] × glucose [mg/dL]/2)]. This formula was initially reported by Simental‐Mendía and colleagues in their seminal work [17]; however, the same authors subsequently clarified that this expression was reported with typos and that the corrected formula is the one we used in the present study [18, 24].

The two formulations can be mathematically rescaled according to the following relationship: TyG (corrected formulation) = TyG (originally reported formulation)/2 + ln(2)/2, where ln(2)/2 ≈ 0.35. Applying this conversion, the thresholds reported by Wei et al. and Unger et al. correspond to TyG values of 4.70 and 4.75, respectively, which are highly comparable with the threshold of 4.78 identified in our cohort.

Notably, our identified discriminatory threshold for diabetes is similar to those reported for diagnosing insulin resistance and metabolic dysfunction in general‐population cohorts. Comparable thresholds calculated with the same formula include 4.68, originally established against the euglycemic‐hyperinsulinemic clamp in a Mexican population [11], 4.49 in an adult population from Maracaibo, Venezuela [25]. This concordance across ethnically and geographically diverse populations suggests that, despite the distinct metabolic and inflammatory milieu of HIV infection, the discriminatory threshold of the TyG index for metabolic risk in PWH does not differ substantially from that established in the general population.

We also tested the predictive value of TyG index for cardiovascular outcomes. We found an incidence rate of CVEs of 6.4 per 1000 persons‐year, that is in line with that reported in a recent metanalysis involving PWH [26]. Higher TyG levels were associated with an increased risk of CVEs, with participants in the highest TyG tertile exhibiting significantly lower event‐free survival and a more than threefold higher adjusted hazard of CVEs compared with those in the lowest tertile.

Montes and colleagues [27] reported that PWH with higher TyG values had more than a 2‐fold increased risk of CVE during follow up, particularly beyond the first 60 months, findings that are consistent with our results. However, the overall incidence of cardiovascular events in their cohort was lower than that observed in our study (3.5 vs. 6.4 per 1000 person‐years). This difference may be partly explained by the substantially shorter follow‐up period (approximately 5 years), which may have limited the opportunity to identify events.

### 4.1 Clinical Implications

Our findings suggest that the TyG index could serve as a valuable and practical tool for clinicians to identify people living with HIV who are at higher risk of developing T2DM. Unlike other indices used to evaluate insulin resistance and T2DM risk, such as HOMA‐IR [28], the TyG index does not require insulin measurement, which is not routinely performed in clinical practice. Instead, it relies on fasting glucose and triglyceride levels, parameters that are readily available and commonly measured. Importantly, the threshold identified in our study (TyG index ≥ 4.78) may offer a simple, actionable cut‐off to guide clinical decision‐making. In individuals exceeding this threshold, targeted interventions, including early lifestyle modification, optimisation of comorbidity management, and more intensive metabolic monitoring, could be implemented promptly to prevent or delay the onset of T2DM. Incorporating the TyG index into routine assessments may thus represent a cost‐effective strategy to improve long‐term metabolic outcomes in this vulnerable population.

Our results are even more important considering that an established CV risk stratification strategy for PWH is not recommended. As such, current Atherosclerotic Cardiovascular Disease (ASCVD) risk equations underestimate CV risk in PWH. The TyG index may be used for rapid large‐scale screening to flag up subjects at higher CV risk.

### 4.2 Strengths and Limitations

A key strength of this study is the inclusion of a large cohort of 477 PWH, who were followed for an extended period of about 14 years. This long‐term follow‐up allowed for the assessment of both metabolic and cardiovascular outcomes over clinically meaningful time horizons, providing robust temporal evidence of the relationship between the TyG index and considered outcomes.

However, our study has some limitations. First, we had no data to compare TyG index with other direct measures of insulin resistance, such as the hyperinsulinemic‐euglycemic clamp or HOMA‐IR, which are considered gold standards for assessing insulin sensitivity. Second, despite adjusting for multiple relevant covariates, we cannot exclude the possibility of residual or unmeasured confounding factors, such as dietary habits, levels of physical activity, or other lifestyle behaviours, which were not systematically assessed but could influence T2DM risk. Third, our study cohort consisted exclusively of Caucasian individuals, which may limit the generalizability of our findings to other ethnic groups with different metabolic profiles or T2DM risk.

In conclusion, in this long‐term cohort of PWH, a higher TyG index was independently associated with an increased risk of developing both T2DM and CVEs. These findings support the use of the TyG index as a simple, low‐cost, and clinically meaningful tool for early metabolic risk stratification in people living with HIV, with potential implications for personalised prevention and management strategies in this high‐risk population.

## Author Contributions

**Daniele Pastori:** conceptualization, Formal Analysis, Writing – original draft; **Gianluca Gazzaniga:** conceptualization, Formal Analysis, Writing – original draft, Visualization; **Tommaso Brogi:** investigation, Data curation, Writing – original draft; **Marco Rivano Capparuccia:** investigation, Data curation, Writing – review and editing; **Caterina Fimiani:** investigation, Writing – review and editing; **Claudio Mastroianni:** investigation, Writing – review and editing; **Ivano Mezzaroma:** conceptualization, Investigation, Writing – review and editing, Supervision.

## Funding

The authors have nothing to report.

## Ethics Statement

The study protocol was approved by the Institutional Review Board at Sapienza University of Rome. The protocol adheres to the ethical guidelines of the Helsinki Declaration.

## Consent

The authors have nothing to report.

## Conflicts of Interest

The authors declare no conflicts of interest.

## Supporting information


**Table S1:** Subjects' demographic and clinical characteristics according to incident Type 2 diabetes mellitus (T2DM).
**Table S2:** Alternative Multivariable logistic regression model for predictors of T2DM with years on antiretroviral therapy as variable instead of Age.
**Table S3:** Subjects' demographic and clinical characteristics according to incident CVEs.

## Data Availability

The data that support the findings of this study are available from the corresponding author upon reasonable request.
