# Supplementary material for: The Triglyceride‐Glucose (TyG) Index and Incident Diabetes and Cardiovascular Events in People Living With HIV‐1 Infection. A 14‐Year Observational Cohort Study
Source: Eur J Clin Invest. 2026 Sep 2;56(9):e70258. doi: 10.1111/eci.70258 (PMC13536519; doi:10.1111/eci.70258)
Supplement: Supplementary file 1 [file ECI-56-e70258-s001.docx]

**The triglyceride-glucose (TyG) index and incident diabetes and cardiovascular events in people living with HIV-1 infection. A 14-year observational cohort study.**

# Supplementary Material

## Supplementary Table 1. Subjects’ demographic and clinical characteristics according to incident type 2 diabetes mellitus (T2DM).

| Characteristic | N | Overall,  N = 477 | No T2DM at FU,  N = 439 | T2DM at FU,  N = 38 | p-value |
| --- | --- | --- | --- | --- | --- |
| Women | 477 | 126 (26%) | 119 (27%) | 7 (18%) | 0.244 |
| Age at enrolment (years) | 477 | 38.0 (31.8, 47.8) | 37.5 (31.4, 47.3) | 43.2 (35.0, 53.4) | 0.011 |
| Years on ART | 457 | 19.0 (11.0, 25.0) | 19.0 (10.0, 25.0) | 22.0 (16.0, 27.0) | 0.007 |
| HCV coinfection | 469 | 110 (23%) | 102 (24%) | 8 (21%) | 0.716 |
| HBV coinfection | 470 | 64 (14%) | 60 (14%) | 4 (11%) | 0.562 |
| CD4 nadir (cells/μL) | 441 | 217.0 (111.0, 345.0) | 222.0 (115.0, 350.0) | 183.0 (92.5, 265.0) | 0.070 |
| BMI (kg/m²) | 424 | 23.7 (22.0, 25.8) | 23.5 (21.8, 25.6) | 25.3 (24.0, 28.4) | <0.001 |
| Hypertension | 477 | 20 (4.2%) | 18 (4.1%) | 2 (5.3%) | 0.668 |
| Dyslipidemia | 477 | 86 (18%) | 78 (18%) | 8 (21%) | 0.613 |
| COPD | 477 | 1 (0.2%) | 1 (0.2%) | 0 (0%) | >0.999 |
| History of stroke or TIA | 476 | 3 (0.6%) | 3 (0.7%) | 0 (0%) | >0.999 |
| History of MI or CHD | 477 | 7 (1.5%) | 7 (1.6%) | 0 (0%) | >0.999 |
| Fasting glucose (mg/dL) | 477 | 88.1 (82.2, 95.5) | 87.0 (82.2, 95.0) | 100.9 (88.1, 113.5) | <0.001 |
| Total cholesterol (mmol/L) | 459 | 4.5 (3.8, 5.4) | 4.5 (3.8, 5.4) | 4.7 (3.9, 5.3) | 0.928 |
| Triglycerides (mg/dL) | 477 | 119.5 (85.0, 172.6) | 118.6 (84.5, 171.2) | 146.5 (107.1, 205.3) | 0.013 |
| TyG index | 477 | 4.6 (4.5, 4.8) | 4.6 (4.4, 4.8) | 4.8 (4.6, 5.0) | <0.001 |
| Creatinine (mg/dL) | 473 | 0.9 (0.8, 1.0) | 0.9 (0.8, 1.0) | 1.0 (0.8, 1.1) | 0.223 |
| Hemoglobin (g/dL) | 395 | 14.3 (13.3, 15.1) | 14.3 (13.2, 15.1) | 14.6 (13.3, 15.4) | 0.648 |
| Platelet count (10^9/L) | 395 | 206.0 (169.0, 244.0) | 208.0 (170.0, 246.0) | 185.0 (160.0, 223.8) | 0.078 |
| White blood cell count (10^9/L) | 396 | 5.59 (4.60, 6.88) | 5.60 (4.62, 6.88) | 5.50 (4.21, 6.79) | 0.455 |
| CD4+ T lymphocytes (%) | 395 | 23.0 (14.0, 31.0) | 24.0 (15.0, 31.0) | 17.5 (12.3, 25.8) | 0.102 |
| CD4+ T lymphocytes (cells/μL) | 395 | 413.0 (238.0, 623.0) | 422.0 (239.0, 629.0) | 330.5 (232.0, 493.5) | 0.229 |
| AST (U/L) | 393 | 25.0 (19.0, 35.0) | 25.0 (19.0, 34.3) | 29.0 (22.0, 46.0) | 0.161 |
| ALT (U/L) | 393 | 24.0 (16.0, 38.0) | 23.0 (16.0, 36.0) | 32.0 (20.0, 53.0) | 0.026 |
| GGT (U/L) | 371 | 24.0 (16.0, 45.5) | 23.0 (16.0, 44.0) | 36.0 (24.0, 58.0) | 0.004 |

*ALT, alanine aminotransferase; ART, antiretroviral therapy; AST, aspartate aminotransferase; BMI, body mass index; CHD, coronary heart disease; COPD, chronic obstructive pulmonary disease; eGFR, estimated glomerular filtration rate; GGT, gamma-glutamyl transferase; HBV, hepatitis B virus; HCV, hepatitis C virus;* *MI, Myocardial Infarction; T2DM, type 2 diabetes mellitus; TIA, transient ischemic attack; TyG, triglyceride-glucose index.*

## Supplementary Table 2: Alternative Multivariable logistic regression model for predictors of T2DM with years on antiretroviral therapy as variable instead of Age.

|  | Univariable Model | | | Multivariable Model | | |
| --- | --- | --- | --- | --- | --- | --- |
| Characteristic | **OR** | **95% CI** | **p-value** | **OR** | **95% CI** | **p-value** |
| High TyG group (vs low)* | 3.57 | 1.82, 7.19 | <0.001 | 2.23 | 1.02, 4.90 | 0.045 |
| Years on ART (per 5-years increase) | 1.33 | 1.09, 1.66 | 0.007 | 1.29 | 1.02, 1.66 | 0.044 |
| BMI | 1.14 | 1.05, 1.24 | 0.002 | 1.14 | 1.05, 1.25 | 0.003 |

**subjects were stratified according to optimal threshold from ROC analysis (TyG=4.78); ART, antiretroviral therapy; BMI, body mass index; TyG, triglyceride-glucose index.*

## Supplementary Table 3. Subjects’ demographic and clinical characteristics according to incident CVEs

| **Characteristic** | **N** | **Overall,** N = 477 | **No CVE,** N = 432 | **CVE,**  N = 45 | **p-value** |
| --- | --- | --- | --- | --- | --- |
| **Female Sex** | 477 | 126 (26%) | 118 (27%) | 8 (18%) | 0.167 |
| **Age at enrolment (years)** | 477 | 38.0 (31.8, 47.8) | 37.7 (31.7, 47.6) | 40.2 (33.2, 49.7) | 0.327 |
| **Years on ART** | 457 | 19.0 (11.0, 25.0) | 19.0 (10.0, 25.0) | 24.0 (18.0, 27.0) | 0.001 |
| **HCV coinfection** | 469 | 110 (23%) | 100 (24%) | 10 (22%) | 0.837 |
| **HBV coinfection** | 470 | 64 (14%) | 60 (14%) | 4 (8.9%) | 0.331 |
| **CD4 nadir (cells/μL)** | 441 | 217.0 (111.0, 345.0) | 220.5 (115.5, 350.0) | 155.0 (70.0, 275.0) | 0.018 |
| **BMI (kg/m²)** | 424 | 23.7 (22.0, 25.8) | 23.7 (22.0, 25.7) | 24.0 (22.5, 26.0) | 0.569 |
| **Hypertension** | 477 | 20 (4.2%) | 15 (3.5%) | 5 (11%) | 0.031 |
| **Dyslipidemia** | 477 | 86 (18%) | 72 (17%) | 14 (31%) | 0.016 |
| **COPD** | 477 | 1 (0.2%) | 1 (0.2%) | 0 (0%) | >0.999 |
| **History of stroke or TIA** | 476 | 3 (0.6%) | 2 (0.5%) | 1 (2.2%) | 0.258 |
| **History of MI or CHD** | 477 | 7 (1.5%) | 3 (0.7%) | 4 (8.9%) | 0.002 |
| **Fasting glucose (mg/dL)** | 477 | 88.1 (82.2, 95.5) | 88.1 (82.2, 95.1) | 90.1 (82.9, 98.0) | 0.188 |
| **Total cholesterol (mmol/L)** | 459 | 4.5 (3.8, 5.4) | 4.5 (3.8, 5.4) | 5.0 (3.7, 5.6) | 0.819 |
| **Triglycerides (mg/dL)** | 477 | 119.5 (85.0, 172.6) | 118.6 (83.6, 164.6) | 147.8 (105.3, 208.0) | 0.007 |
| **TyG index** | 477 | 4.6 (4.5, 4.8) | 4.6 (4.4, 4.8) | 4.8 (4.6, 4.9) | 0.003 |
| **Creatinine (mg/dL)** | 473 | 0.9 (0.8, 1.0) | 0.9 (0.8, 1.0) | 0.8 (0.8, 1.0) | 0.256 |
| **Hemoglobin (g/dL)** | 395 | 14.3 (13.2, 15.1) | 14.4 (13.3, 15.1) | 13.8 (13.0, 15.8) | 0.819 |
| **Platelet count (10^9/L)** | 395 | 206.0 (169.0, 245.0) | 206.5 (169.0, 242.0) | 196.0 (165.0, 252.0) | 0.879 |
| **White blood cell count (10^9/L)** | 396 | 5.59 (4.60, 6.88) | 5.55 (4.61, 6.81) | 6.10 (4.31, 6.95) | 0.809 |
| **CD4+ T lymphocytes (%)** | 395 | 23.0 (14.0, 31.0) | 23.0 (15.0, 31.0) | 21.0 (11.0, 28.0) | 0.161 |
| **CD4+ T lymphocytes (cells/μL)** | 395 | 413.0 (237.0, 624.0) | 421.0 (239.0, 629.0) | 334.0 (191.0, 540.0) | 0.201 |
| **AST (U/L)** | 393 | 25.0 (19.0, 35.0) | 25.0 (19.0, 34.0) | 27.0 (22.5, 44.5) | 0.066 |
| **ALT (U/L)** | 393 | 24.0 (16.0, 38.0) | 23.0 (16.0, 38.0) | 33.0 (21.0, 49.5) | 0.054 |
| **GGT (U/L)** | 371 | 24.0 (16.0, 46.0) | 23.0 (16.0, 44.0) | 34.0 (22.0, 96.0) | 0.007 |

*ALT, alanine aminotransferase; ART, antiretroviral therapy; AST, aspartate aminotransferase; BMI, body mass index; CHD, coronary heart disease; COPD, chronic obstructive pulmonary disease; CVE, Cardiovascular Event; GGT, gamma-glutamyl transferase; HBV, hepatitis B virus; HCV, hepatitis C virus;* *MI, Myocardial Infarction; TIA, transient ischemic attack; TyG, triglyceride-glucose index.*
